# Comparative Study of Polycaprolactone Electrospun Fibers and Casting Films Enriched with Carbon and Nitrogen Sources and Their Potential Use in Water Bioremediation

**DOI:** 10.3390/membranes12030327

**Published:** 2022-03-15

**Authors:** Daniella Alejandra Pompa-Monroy, Ana Leticia Iglesias, Syed Gulam Dastager, Meghana Namdeo Thorat, Amelia Olivas-Sarabia, Ricardo Valdez-Castro, Lilia Angélica Hurtado-Ayala, José Manuel Cornejo-Bravo, Graciela Lizeth Pérez-González, Luis Jesús Villarreal-Gómez

**Affiliations:** 1 Facultad de Ciencias de la Ingeniería y Tecnología, Universidad Autónoma de Baja California, Tijuana 21500, Baja California, Mexico; daniella.pompa@uabc.edu.mx (D.A.P.-M.); aiglesias@uabc.edu.mx (A.L.I.); perez.graciela@uabc.edu.mx (G.L.P.-G.); 2 Facultad de Ciencias Químicas e Ingeniería, Universidad Autónoma de Baja California, Tijuana 22260, Baja California, Mexico; lilyhurtado@uabc.edu.mx (L.A.H.-A.); jmcornejo@uabc.edu.mx (J.M.C.-B.); 3 National Collection of Industrial Microorganism (NCIM), CSIR-National Chemical Laboratory, Pune 41008, Maharashtra, India; sg.dastager@ncl.res.in (S.G.D.); mn.thorat@ncl.res.in (M.N.T.); 4 Centro de Nanociencias y Nanotecnología, Universidad Nacional Autónoma de México, Ensenada 22860, Baja California, Mexico; ameliaolivas610@gmail.com (A.O.-S.); ricardovc1030@gmail.com (R.V.-C.)

**Keywords:** electrospinning, poly (caprolactone), bacterial growth, carbon source, nitrogen source

## Abstract

Augmenting bacterial growth is of great interest to the biotechnological industry. Hence, the effect of poly (caprolactone) fibrous scaffolds to promote the growth of different bacterial strains of biological and industrial interest was evaluated. Furthermore, different types of carbon (glucose, fructose, lactose and galactose) and nitrogen sources (yeast extract, glycine, peptone and urea) were added to the scaffold to determinate their influence in bacterial growth. Bacterial growth was observed by scanning electron microscopy; thermal characteristics were also evaluated; bacterial cell growth was measured by ultraviolet-visible spectrophotometry at 600-nm. Fibers produced have an average diameter between 313 to 766 nm, with 44% superficial porosity of the scaffolds, a glass transition around ~64 °C and a critical temperature of ~338 °C. The fibrous scaffold increased the cell growth of *Escherichia coli* by 23% at 72 h, while *Pseudomonas aeruginosa* and *Staphylococcus aureus* increased by 36% and 95% respectively at 48 h, when compared to the normal growth of their respective bacterial cultures. However, no significant difference in bacterial growth between the scaffolds and the casted films could be observed. Cell growth depended on a combination of several factors: type of bacteria, carbon or nitrogen sources, casted films or 3D scaffolds. Microscopy showed traces of a biofilm formation around 3 h in culture of *P. aeruginosa*. Water bioremediation studies showed that *P. aeruginosa* on poly (caprolactone)/Glucose fibers was effective in removing 87% of chromium in 8 h.

## 1. Introduction

Electrospinning is a widely used technique in various branches of biomedical research, focusing mainly on tissue engineering, where it is used to prepare scaffolds that mimic a synthetic extracellular matrix, which can be used for nerve regeneration [1], osteoblast culture [2], vascular grafts [3], regeneration of muscle cells [4] and dermal dressings [5,6]. It is a technique used to produce fine fibers from polymer solutions, either from natural or synthetic sources [7,8,9]. The process can be controlled using a variety of collectors or by modifying the parameters with which the material is produced, creating a three-dimensional structure known as scaffolding [10]. However, its use in biotechnology has been very limited; electrospun nanofibers are mainly being used as filters, to avoid contamination from bacteria infiltration thanks to the specific porosity in the general mat [11]. 

The interest to include bacteria in electrospun fibers for biotechnological application is not new; *Escherichia coli* and *Streptomyces albus* have been loaded in electrospun fibers. The bacteria survived the electrospinning process, where *E. coli* showed a better viability than *S. albus* [12]. Other bacteria such as *Bacillus animalis lactis* Bb12 (present in the human intestine and auxiliary in the absorption of lactose) survive without any problem using poly (vinyl alcohol) (PVA); in this manner, the bacteria can be preserved inside the fibers for up to 130 days at 4 °C [13]. These containment modalities have been tested within bioreactors using *Pseudomonas fluorescens, Zymomonas mobilis* and *E. coli*; in this case, *P. fluorescens* and *E. coli* did not survive the process, though *Z. mobilis* remained viable in 93%; like *B. animalis lactis*, it could be preserved and used for fermentation [14]. In other applications, the fibers have been used to improve the efficiency of bacterial cell growth to promote plant growth. For example, *Bradyrhizobium japonicum* and *Bradyrhizobium elkanii*, which are present in the rhizosphere of the soybean root and help nitrogen fixation, were able to improve plant growth in soil with adverse conditions by using a water-retaining polymer [15].

Many factors affect bacterial adhesion, such as surface charge, surface energy, substrate characteristics (leading to the formation of biofilms), membrane composition and surface elements, such as pili or fimbriae, as well as steric factors, among others [16]. Recently, attempts have been made to assess whether these factors have an effect on bacterial reproduction when electrospun fibers are present in the culture media. In 2017, three bacterial strains were cultivated, *Micromonospora* sp., *Streptomyces* sp. and *E. coli*. In the presence of poly (acrylic acid) (PAA) and alginate fibers, the concentration of *Streptomyces* sp. increased by 10%, when compared against the cell suspension with normal growth (control). In the case of *Micromonospora* sp. and *E. coli*, no improvements were observed [17]. In the same year, Moffa et al. [18] carried out tests to verify if nanofibers helped the production of secondary metabolites obtained from *Streptomyces lividans*; in this instance, a 50% increment in bacterial concentration was observed; the latter can be attributed to the ability of *S. lividans* to generate mycelia (which is the source of secondary metabolites), which would be favored by using scaffolding as an anchor site, thus reducing the time of adaptation of the bacteria. In industry, biofilms have an ambivalent role, where they can be both beneficial and harmful. A biofilm can affect an industrial process by its structure, its metabolism or its resultant products, i.e., bacteria [19,20,21]. When they work for our benefit, they can be used for water treatment [22] or various types of solid fermentation [23,24]. On the other hand, the formation of biofilms allows the sessile bacteria to form it, horizontally exchanging genetic information and increasing their resistance to both antimicrobial agents and physical removal methods such as ultraviolet light. Moreover, these high rates of genetic exchange, alteration in biodegradation and an increase in the production of metabolites eventually lead to a decrease in efficiency and damage to equipment, pipe obstruction and reduction in heat exchange systems [25,26].

*Pseudomonas* strains are important for the bioremediation of contaminated oil spills and heavy metal in water sources, which represents one of the great threats to environmental pollution. Additionally, the ability of the bacteria to form biofilms, which improves their survival in nutrient-stressed conditions or change of cycle of attachment, as well as the release of biofilm-associated cells, can, in conjunction with the natural properties of the fibrous scaffolds, benefit the bioremediation process [27]. Furthermore, one of the most studied and reported biofilm formations is the one produced by *Escherichia coli* in view of its potential in biotechnological application, due to its well-known genetic content [28]. *E. coli* has been used in the bioremediation of the insecticide methomyl [29] and waste hydrocarbons [30], amongst others. Finally, *Staphylococcus aureus* has been reported to have a great potential in heavy metal removal of zinc, chrome, cadmium and nickel [31]. Moreover, *Staphylococcus aureus* can also be useful for oil bioremediation in vat dyes, textile effluents, etc. [32].

Poly (caprolactone) (PCL) is a synthetic biodegradable polymer that has a wide range of uses, especially in tissue engineering [19]. This crystalline polyester has a high ductility due to its low glass transition temperature (−60 °C); PCL is considered an ecological material that can be used in disposable materials for the medical, food and agricultural industries [20]. However, the use of this polymer with different sources of carbon and nitrogen as a promoter for bacterial growth has not been studied. Finally, the fields of biomedicine, biotechnology, genomics and proteomics are in search of new materials that can lead to the development of biocompatible artificial tissues, organs, stem cells or nanopharmaceuticals for the controlled dosage of drugs. Thus, electrospun nanofibers can be used for the overproduction of microbial biomass, and/or biofilm production for biotechnological applications, such as in bioreactors for industrial use, where the sterility of media and equipment could be guaranteed, since the scaffolds can be autoclaved at 121 °C without losing their properties. Since most fermentations take place within the range of 30 to 37 °C, stability is also guaranteed during this process [12,17,28].

Therefore, in this work we evaluated the effect of PCL scaffolding and cast films on bacterial growth by adding different carbon (glucose, fructose, lactose and galactose) and nitrogen sources (peptone, extract of yeast, glycine and urea), and furthermore the physicochemical change that occurs in these enriched fibers and how bacterial colonies evolve in these scaffolds is also studied. In the present study, electrospun nanofibers were used to determine their efficiency as a growth accelerator through the formation of biofilms in bacteria of biotechnological interest. Lastly, the use of PLC/Glu fibers for the bioremediation of Cr contaminated water was studied.

## 2. Materials and Methods

### 2.1. Materials

For the cell growth assay, the bacterial strains *Staphylococcus aureus* (ATTCC 25923), *Escherichia coli* (ATTC 25922) and *Pseudomonas aeruginosa* (ATCC 27853) were used. Solvents such as methanol ACS (Fermont), Tetrahydrofuran (THF) (Tecsiquim) are analytical grade. Poly (ε-caprolactone) (average MW ~ 80,000 g/mol, Sigma Aldrich, St. Louis, MO, USA). All carbon and nitrogen sources, including glucose, fructose, galactose, lactose, peptone, glycine, yeast extract and urea, were obtained from Sigma Aldrich and used as received.

### 2.2. Preparation of Polymeric Blends

Carbon and nitrogen sources solutions (described in Table 1) were prepared by dissolving 0.16% of the corresponding source in methanol and stirred for 2 h at 50 °C. Afterwards, a 13% solution of PCL in THF was added to the blend and dissolved by magnetic stirring at 500 rpm for 5 h at 50 °C.

### 2.3. Films and Fibers

#### 2.3.1. Electrospinning

PCL solutions and those with *carbon and nitrogen sources* (PCL/Cs and PCL/Ns) were electrospun with the following conditions: 20 kV for one hour, with a distance of 18 cm between an aluminum-coated metal collector and the syringe needle (21G), a flow of 3 mL/h, a temperature in the range of 25–32 °C and a relative humidity of 30–50%.

#### 2.3.2. Film Production

In order to have a flat structure to compare the efficiency of the fibers, Cs and Ns films were produced by pouring 2 mL of the polymeric blends on 35 mm petri dishes, and letting the solvents evaporate overnight in a fume hood.

### 2.4. Fourier Transform Infrared Spectroscopy (FTIR)

To observe whether chemical changes occurred after material processing through electrospinning and observed the presence of Cs and Ns, fibers were evaluated by infrared spectrum. Samples were taken from each scaffold with dimensions of 0.5 × 0.5 cm and placed on the lens of the ATR module. Samples were analyzed in a spectrum from 4000–400 cm^−1^ wavenumbers. The spectra were analyzed with Omnic software.

### 2.5. Differential Scanning Calorimetry (DSC) and Thermogravimetric Analysis (TGA)

For the thermogravimetric analysis, 5 mg samples were placed in platinum trays, which were heated from room temperature (about 25 °C) to 600 °C with a heating ramp of 10 °C/min under a nitrogen atmosphere. In the case of differential scanning calorimetry, samples were placed in platinum trays that were subsequently pressed and sealed. The samples were processed with the same characteristics of the thermogravimetric analysis. The thermograms were analyzed with TA universal analysis software.

### 2.6. Culture Medium Preparation, Inoculation and Adjustment

Bacterial strains such as *Escherichia coli* (ATCC 25922), *Pseudomonas aeruginosa* (ATCC 27853) and *Staphylococcus aureus* (ATCC 25923) were cultivated in nutrient broth No. 1 (Sigma Aldrich) and Minimal Salt Broth (MSB) (Sigma Aldrich). The broths were dissolved in distilled water in 25 mL Erlenmeyer flasks (one for each bacteria strain) and sterilized at 121 °C for 15 min. The cryopreserved strains (0.5 mL) were cultivated in each prepared media, and incubated for 18 h at 35 °C. The obtained bacterial suspensions were used to subsequently take aliquots, which were adjusted in nephelometers tubes to 0.5 McFarland with sterile saline water.

### 2.7. Bacterial Cell Growth Assay

To test the effect of the fibers on bacterial cultures, samples of fibers and films of 5 mm-diameter were placed in triplicate on a sterile 96-well plate; 150 µL of sterile nutrient broth and 50 µL of cell suspension of each bacteria strain, adjusted to 0.5 of the McFarland nephelometer, were added. As a negative control of bacterial growth, an inoculated medium was used, whilst gentamicin (10 mg/mL) was used as a positive control. The exposed bacteria (*Escherichia coli, Staphylococcus aureus* and *Pseudomonas aeruginosa)* were incubated at 37 °C for 24, 48 and 72 h, and afterwards absorbance was measured in a Microplate reader (Thermo Scientific) at 600 nm. Cell culture concentration was determined using Equation (1).

(1) $$\%\;\mathrm{cell}\;\mathrm{growth}=\frac{\mathrm{Sample}\;\mathrm{optical}\;\mathrm{density}\;}{\mathrm{Negative}\;\mathrm{control}\;\mathrm{optical}\;\mathrm{density}}\times 100\;\%$$

### 2.8. Biofilm Formation Observation by Scanning Electron Microscopy (SEM)

The morphology and diameter of the polymeric fibers were analyzed by a field emission microscope JEOL JSM 7600F (JEOL Ltd., Tokyo, Japan). To obtain the images, a voltage of 20 kV and a gold coating were used. The diameter of the fibers and superficial porosity of the scaffolds of the selected samples were measured using Image J software, with 30 measurements of at least two different images of each sample (4000× and 6000×). In order to show an example of bacterial growth in the 3D fibers, a *P. aeruginosa* suspension was cultivated for 3 and 6 h in MSB in PCL/Glu scaffolds, and a sample submerged for 3 h with just media was also prepared as a control. Since the samples were not conductive, they required prior preparation with a thin gold layer. The scaffolds were recovered and placed on slides previously bathed in a 0.1% gelatin solution. Moreover, the samples were dried at room temperature for 24 h. Then, in a metal sample holder, a piece of graphite tape was placed. A small portion of the cut samples were placed on the graphite tape, and samples were coated with a thin layer of metallic gold by plasma-assisted cathodic pulverization, at a voltage of around 1 kV for 2 min. After this, the samples were placed in a tray and the micrographs images were obtained from different areas at different amplification magnitudes (2000, 4000 and 6000×), and pristine *P. aeruginosa* cells images were taken separately [6].

### 2.9. Chromium Removal Assay

In order to demonstrate the effectiveness of the bacterial biofilms derived from the electrospun matrices, *Pseudomonas aeruginosa* was selected from the studied bacterial strains to be evaluated in the bioremediation of chromium from contaminated water. Analogously, PCL/Glu fibers were selected as a base for biofilm formation. For the study, the *Pseudomonas* strain was cultured in Luria Bertani (LB) broth, containing Cr (VI) solution (0.5 mg/mL). The culture was incubated on a rotary shaker at 37 °C for 24 h. Every 4 h, the culture was centrifuged (10,000 rpm, 10 min). The culture medium without the chromium salt was used as the negative control. In this assay, *P. aeruginosa* reduced the Cr (VI) ion to Cr (III) ion, and this was later quantified. The reduced Cr (III) content of the cell-free supernatant was detected using a UV/Vis spectrophotometer DU^®^730, Beckman Coulter at 565 nm. Furthermore, the optical density of the bacterial isolates was registered individually, to monitor the growth rate of bacteria in relation to chromium salt removing efficiency. Measurements were taken after 4, 8, 12 and 24 h of biofilm incubation. A total of 3 replicates for each group of samples (PCL/Glu fibers, PCL/Glu films and pristine bacteria) were used to carry out this test; each replicate was contained in individual flasks (250 mL) containing the contaminated LB broth (100 mL). In order to estimate the Cr (III) concentration, a standard curve was developed in a LB broth containing the chromium salt at different concentrations (0.05, 0.1, 0.20, 0.4, 0.6, 0.8 and 1 mM). The percentage of Cr removal was obtained using the following formula (Equation (2)):

(2) $$\%\;\mathrm{Chromium}\;\mathrm{removal}=\frac{\mathrm{Samples}\;\mathrm{optical}\;\mathrm{density}\;}{\mathrm{Control}\;\mathrm{optical}\;\mathrm{density}}\times 100\;\%$$

### 2.10. Statistical Analysis

Bacterial cell growth assay and chromium removal assay were done in triplicate. These results were reported as mean and standard deviation, and the data was processed by the one-way analysis of variance (ANOVA) using the Graph Pad Prism version 6.0c software. The results were considered statistically significant when *p* < 0.05. 

## 3. Results

### 3.1. Fourier Transform Infrared Spectroscopy (FTIR)

**Figure 1:**
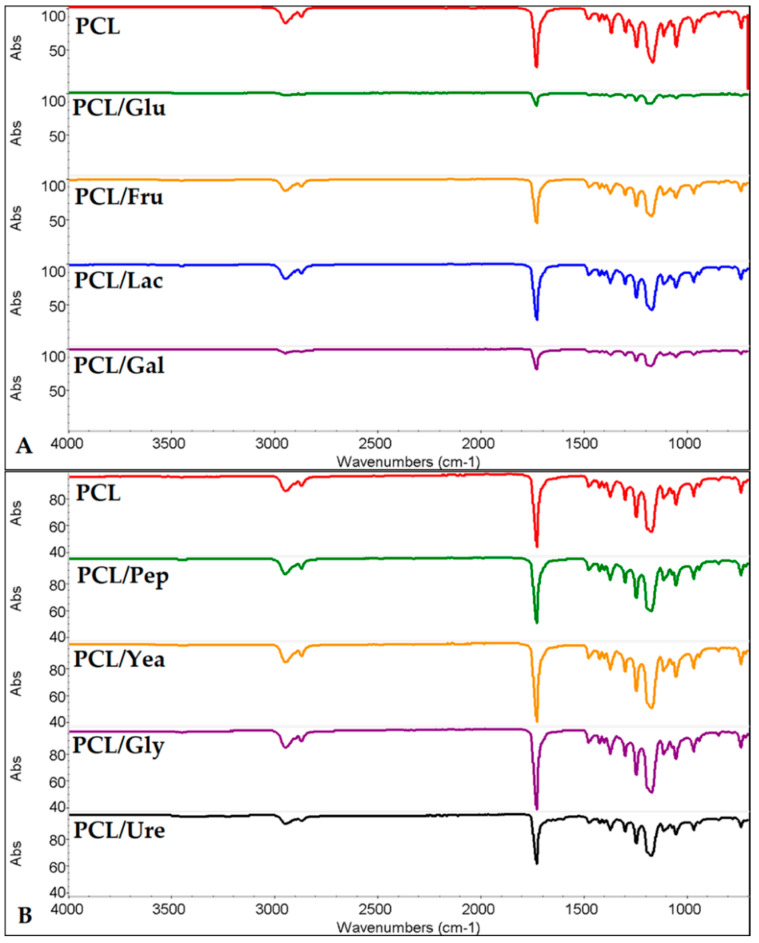
Infrared spectrum of PCL with (**A**) PCL/Cs fibers (**B**) PCL/Ns fibers.

The infrared spectra of PCL and PCL with carbon sources (PCL/Cs) are shown in Figure 1A. For the PCL control, the terminal -CH_3_ and the -CH_2_ alkyl groups present absorption peaks ν(C-H) (in red) at 2867 cm^−1^ and 2942 cm^−1^, respectively. The characteristic peak for the C=O group can be observed at 1724 cm^−1^, and the stretching vibration for the RCOOR’ group at 1160 cm^−1^; similar peaks have been reported earlier [33]. For the PCL-enriched monosaccharides and disaccharides fibers, given the small amount of sugar within the PCL solution, the equipment may not be able to detect differences in the spectrum. The same phenomenon may occur in the PCL spectrum with nitrogen sources (PCL/Ns) (Figure 1B). There is no evidence that indicates the presence of another substance or the modification of the polymer. 

### 3.2. Differential Scanning Calorimetry (DSC) and Thermogravimetric Analysis (TGA)

**Figure 2:**
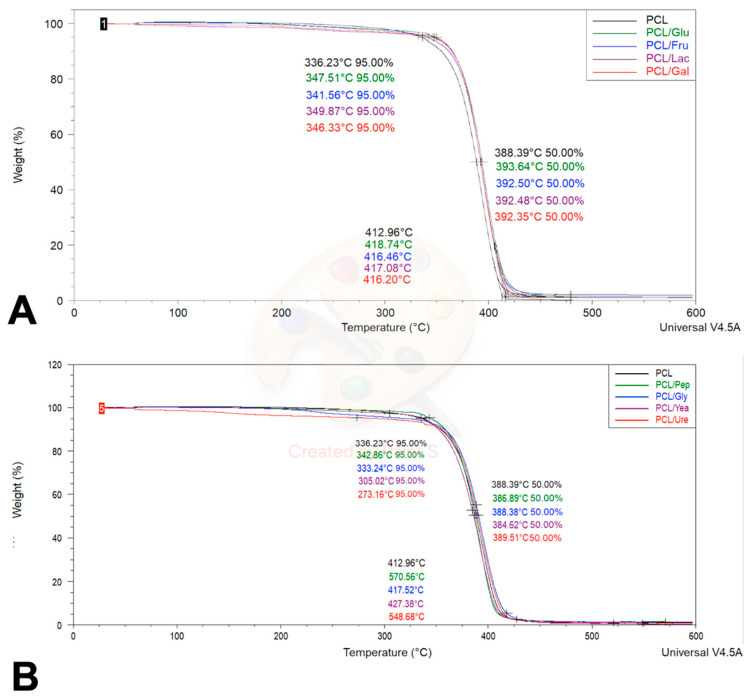
TGA thermogram regarding the weight percentage changes (**A**) PCL/Cs fibers, (**B**) PCL/Ns fibers.

The thermogravimetric analysis of the PCL and PCL/Cs fibers is observed in Figure 2A and Table 2. The PCL sample begins its degradation stage at 336.23 °C, and reaches a critical degradation temperature at 388.39 °C; a behavior previously described by several authors [19,34,35]. Although the phase changes are barely visible, given the amount of sugar added to the sample, its effects can be seen due in the change in degradation temperature; in general, the temperature increased with incorporation of saccharide. In the PCL/Glu fiber, for example, the disaccharide increased the critical degradation temperature by 5.25 °C, followed by the monosaccharide’s scaffolds PCL/Fru with 4.11 °C, PCL/Lac fibers with 4.09 °C and PCL/Gal scaffolds with a 3.96 °C increase. 

On the other hand, Figure 2B and Table 2 show the thermograms of the PCL/Ns fibers. As in sugars, nitrogen sources also change phase and degradation temperatures, but follow a different type of behavior when compared to PCL/Cs fibers. The PCL/Ure sample increased the critical degradation temperature by 1.12 °C, while the presence of glycine in PCL/Gly sample did not significantly alter its critical degradation temperature with a variation of 0.01 °C, while in contrast, for PCL/Yea scaffolds, the temperature decreased by 3.7 °C, followed by PCL/Pep fibers with 1.5 °C.

**Figure 3:**
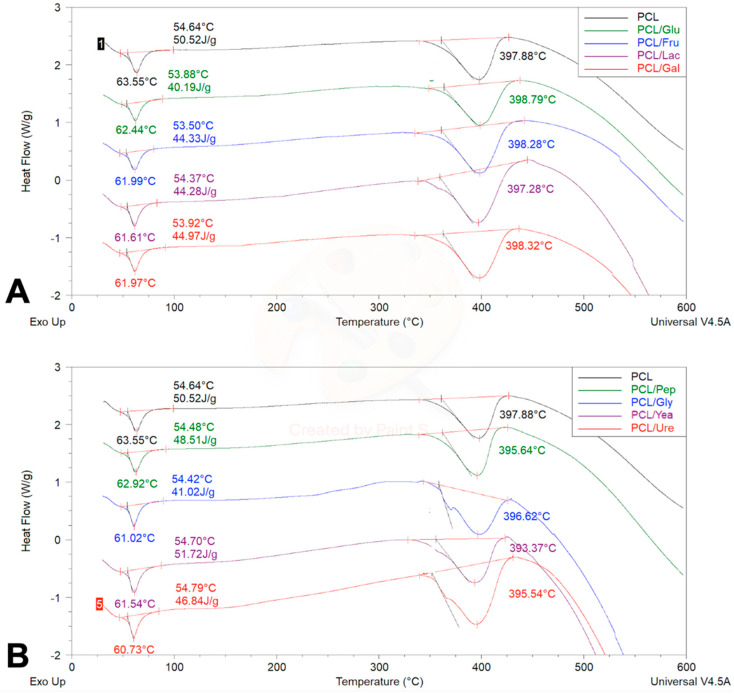
DSC thermogram for PCL fibers. (**A**) PCL/Cs and (**B**) PCL/Ns fibers.

In the case of differential scanning calorimetry results, Figure 3A and Table 3 show the thermograms of the PCL and PCL/Cs samples. As previously reported [33], PCL has a melting point around 60 °C, and according to the thermogram the PCL control has a melting point of 63.55 °C; the addition of carbon sources reduces this value to a range between 61.61 °C and 62.44 °C, possibly indicating not only the melting of the material, but also Cs present in the fibers, given the low temperatures at which these phenomena occur. The next relevant event in the thermogram arises at ~398 °C, indicating the decomposition phase of the material, which has already been reported [35]. Figure 3B and Table 3 show the thermograms of the PCL and PC/Ns samples. As with the samples with carbon sources, the presence of nitrogen reduces the melting temperature of the material to within a range of 60.73 °C and 62.92 °C. The PCL/Ure sample is the fastest to reach the decomposition phase, at 395.54 °C, followed by PCL/Yea fibers at 393.37 °C, PCL/Gly fibers at 396.62 °C and finally PCL/Pep mats at 395.64 °C.

### 3.3. Bacterial Cell Growth Assay

**Figure 4:**
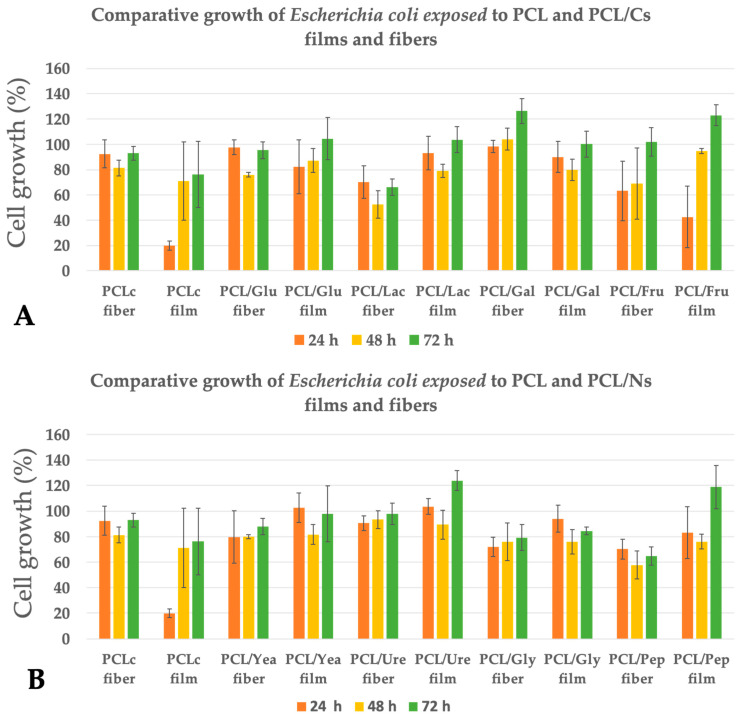
Comparative growth of *Escherichia coli* exposed to (**A**) PCL/Cs fibers and films and (**B**) PCL/Ns fibers and films.

In this assay, in order to evaluate if bacterial cell growth was promoted by 3D scaffolds or by cast films, PCL/Cs and PCL/Ns fibers and films with the same chemical formulation were prepared. For all the studies, normal bacterial growth was taken as the negative control and gentamicin as positive control. Figure 4 shows the cell growth of *Escherichia coli* exposed to the PCL/Cs and PCL/Ns fibers and films at 24, 48 and 72 h. In general, when compared to the normal growth of *E. coli* in the case of PCL/Cs samples, no significant increase in growth can be seen, with the exception of PCL/Gal fibers and PCL/Fru films with a ~26% and ~23% increase cell growth after 72 h of incubation, respectively. For the PCL/Ns samples, PCL/Ure films and PCL/Pep films increased cellular growth by approximately ~20%. The rest of PCL/Cs and PCL/Ns fibers and films were non-toxic, with exception of the PCL control films which diminished bacterial growth, but non-toxic when the PCL was electrospun. In every instance, the highest % of cell growth was seen after 72 h of incubation. No clear cell growth tendency can be observed between days (ANOVA *p* < 0.05). Moreover, when comparing PCL/Cs and PCL/Ns fibers against PCL/Cs and PCL/Ns films, the structure (film of fibers) appears to have no effect in the growth of *E. coli* (ANOVA *p* < 0.05).

**Figure 5:**
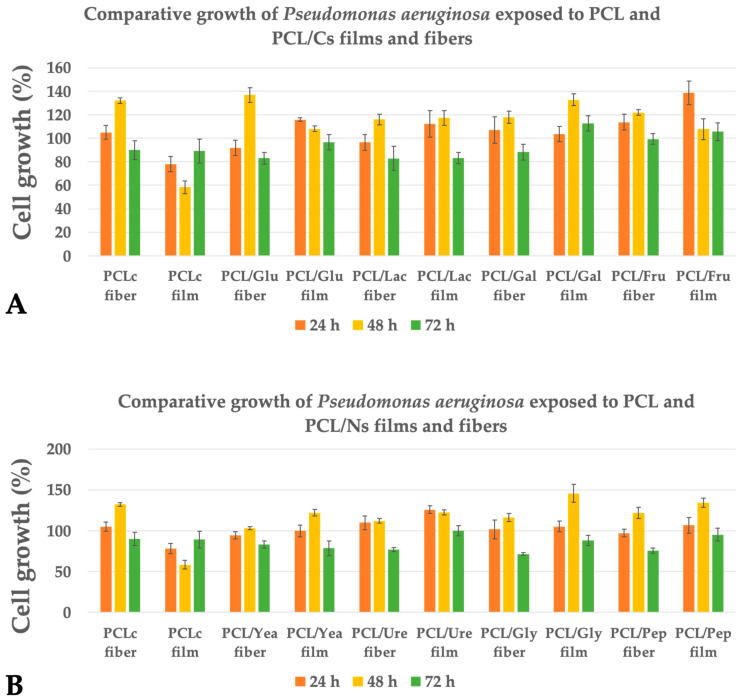
Comparative growth of *Pseudomonas aeruginosa* exposed to (**A**) PCL/Cs and (**B**) PCL/Ns for fibers and films.

**Figure 6:**
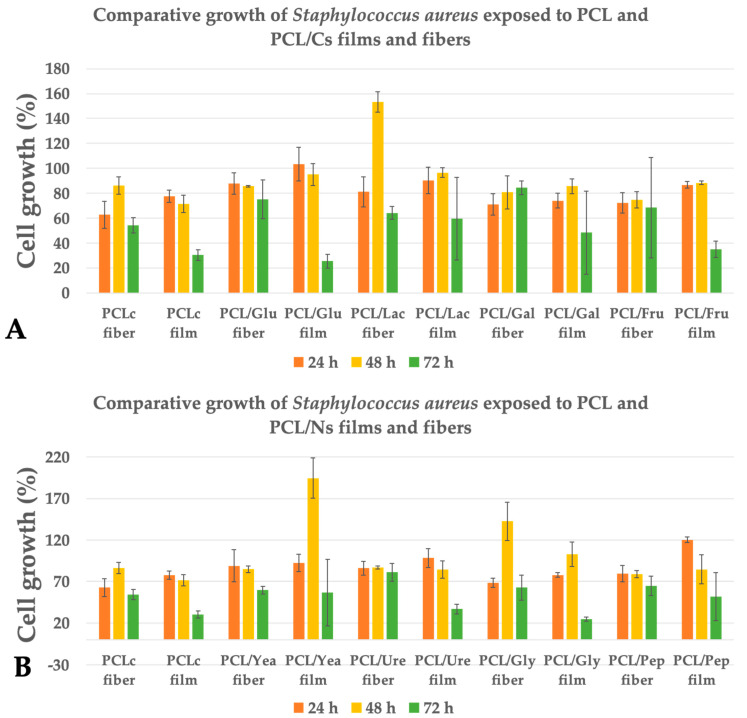
Comparative growth of *Staphylococcus aureus* exposed to (**A**) PCL/Cs and (**B**) PCL/Ns for fibers and films.

While in *E. coli*, an increase in bacterial growth was not observed, both *P. aeruginosa* (Figure 5) and *S. aureus* (Figure 6) showed an improvement in the performance of the culture. In the case of *P. aeruginosa*, PCL/Gly film presented the highest growth stimulation of up to ~46% at 48 h, followed by PCL/Fru film (~39% at 24 h) and PCL/Glu fiber (~37% at 48 h), respectively. Depending of the incubation day tested, most of the PCL/Cs and PCL/Ns fibers and films have shown an improvement in *P. aeruginosa* growth (ANOVA *p* < 0.05), with exception of the pristine PCL film. No clear advantage of the 3D fibrous structure can be seen compared to their respective films (ANOVA *p* < 0.05). In most of the samples, after the 48 h of incubation, the highest cell growth rate was observed.

Lastly, *S. aureus* was the strain that showed the best results in terms of increased bacterial concentration at 48 h. Upon contact with the PCL/Yea films, the concentration increased by ~95% compared to the control culture, followed by PCL/Lac and PCL/Gly fibers, which increased the cell growth by ~53% and ~42%, respectively. Despite the latter good results, the rest of samples did not significantly increase the growth of *S. aureus* (ANOVA *p* < 0.05).

### 3.4. Biofilm Formation Observation by Scanning Electron Microscopy (SEM)

PCL and PCL/Cs and PCL/Ns fibers and films samples were prepared, the obtained micrographs are presented in Figure S1. Fiber diameter was calculated with the software image J, using 30 measurements per field, obtaining the mean fiber diameter and standard deviation (SD). As observed in Figure S1, some defects and beads were found; possibly due to the interaction between the polymer molecules in the solvent and the electrical forces that are not strong enough to attract the excess of ions that are carried out with the polymeric jet stream, which is formed thanks to the electrical field created by the voltage. This disequilibrium provoked the cylindrical jet to become a series of droplets, which solidified into beads, supported on the nanofibers [36,37]. Nonetheless, fibers are randomly deposited, creating the desired tridimensional network for bacterial penetration. The poor quality of the micrographs in Figure S1 is due to technical problems of the equipment used (cross-sectional black and white lines).

Fiber diameters for every sample were obtained in the range of 313 to 766 nm. PCL/Glu, PCL/Lac and PCL/Ure were the fibrous matrices with thinnest fibers, with the smallest fiber diameter corresponding to the disaccharide PCL/Lac sample 301 ± 072 nm; interestingly, the latter samples possess the lowest standard deviation, denoting more reproducibility and homogeneity amongst the fibers. On the other hand, the sample with thickest fibers was the PCL/Yea sample with 766 ± 290 nm, which was also the highest standard deviation for all samples. The rest of the samples were in the range between 400 and 500 nm (Table 4). No other fiber diameter pattern can be observed in the scaffolds, aside from those mentioned above.

**Figure 7:**
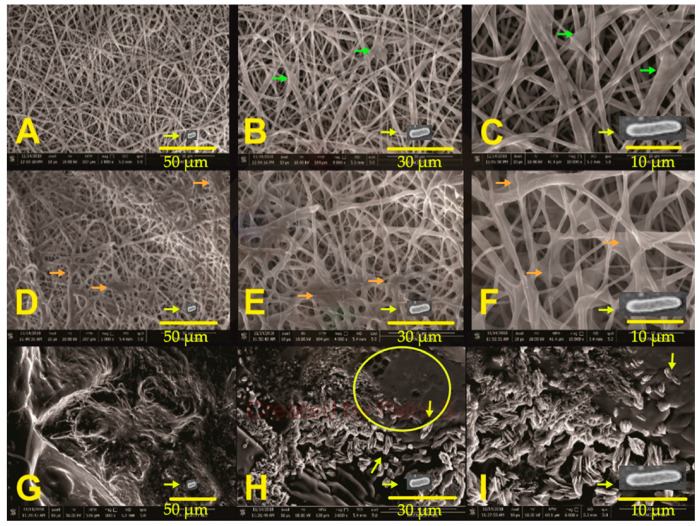
SEM micrograph of PCL/Glu fibers. First line (**A**–**C**) PCL/Glu fibers at 2000, 4000 and 6000× without inoculum after 6 h on MSB; second line (**D**–**F**) 2000, 4000 and 6000× after 3 h of growth with *P. aeruginosa;* and third line (**G**–**I**) 2000, 4000 and 6000× after 6 h of growth with *P. aeruginosa* on MSB.

Based on the bacterial cell growth assay results (*vide supra*), PCL/Glu fibers were chosen to observe biofilm formation and to evaluate its efficacy, in promoting heavy metal removal from contaminated water (using LB media as a model in the assay). PCL/Glu fibers have a fairly uniform appearance when submerged in just MSB media (Figure 7A). In certain points, the fibers change their diameters and present slight defects in their morphology. A closer look at the material shows that the defects are due to melting of the polymer (green arrows), possibly due to changes in the environment and transport of the material (Figure 7B,C) after 6 h in the MSB media. The average fiber diameters were in the range of 313 ± 089 nm, with a superficial porosity percentage of the whole scaffolds of approximately 44.31%. 

Figure 7D shows that for fibers in *P. aeruginosa* bacterial culture with MSB, after three hours of incubation, a covering begins to develop on PCL/Glu pores and binds the fibers, possibly indicating the development of biofilm on the scaffold (orange arrows). When evaluating the images at 4000× and 6000× (Figure 7E,F), the coating appears translucent and amorphous. In the upper part of Figure 7F, an irregular formation in layers on the fiber can be observed; this behavior is similar to that observed in Figure 7E, where clusters on the fibers shaped like small spheres can be observed (orange arrows).

After six hours, the scaffold has been consumed by the biofilm, and only the thickest fibers appear to remain on the surface. The morphology of the film is interesting, as the biofilm penetrates and grows inside the 3D fibrous structure. In Figure 7G,I, an irregular topology was observed, in which no trace of the fibers can be observed, and crystal formations can be seen—most likely salts of the medium in which the fibers were submerged.

Finally, a *Pseudomonas aeruginosa* cell image was added and overlapped on the micrographs of Figure 7; this image was obtained separately, and the size of the cell adjusted to ~10 μm (the reported bacteria size) [38]. As observed, *P. aeruginosa* cells are small enough to penetrate into the 3D fibrous scaffolds and replicate between the fibers. Some similar *bacillus* shape structures (yellow arrows) can be observed in Figure 7H,I, giving evidence of the bacterial presence and biofilm formation. Moreover, a biofilm layer can clearly be noticed over the fibers in Figure 7H (yellow circle).

### 3.5. Chromium Removal Assay

As an example of the potential for bioremediation of contaminated water with heavy metals, PCL/Glu fibers and films were chosen to produce *P. aeruginosa* biofilms, in order to evaluate if the fibers presence enhances the chromium removal, using microbiological media (LB) as an example of contaminated water. These fibers were chosen due to their capacity to promote the cell growth of *P. aeruginosa*, which is a well-known microorganism for the removal of heavy metals in water [39]. For the bioremediation study, Luria Bertani (LB) broth media was contaminated with Cr (VI) solution (0.5 mg/mL). A pristine *P. aeruginosa* suspension was employed as a control and bioremediation agent. Additionally, *P. aeruginosa* biofilms were cultivated in PCL/Glu films and PCL/Glu fibrous scaffolds. The study was initially planned for 24 h, but after 12 h, no presence of chrome was detected. The percentage weight loss vs. time was recorded. 

**Figure 8:**
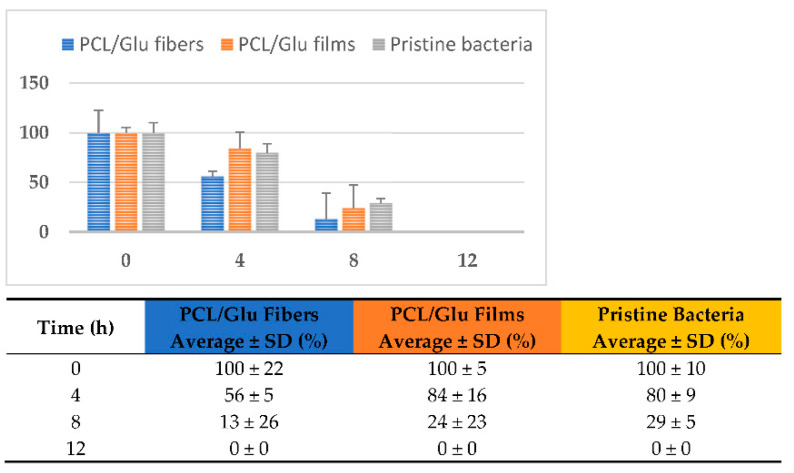
Percentage (%) of chromium uptake by *P. aeruginosa* in water bioremediation assay.

In general, as observed in Figure 8, the 3D structure of the PCL/Glu formulation fibers accelerated the bacterial metabolism uptake of chromium after just 4 h of incubation, with almost 50% removal of the total mass, while PCL/Glu films and the pristine bacterial suspension degraded only 20% of the metal salt mass. After 8 h of incubation, almost 90% of the metal was metabolized by the bacteria in the reaction containing PCL/Glu fibers, whereas just 70–80% of mass loss was achieved with the pristine bacteria and PCL/Glu films, respectively. After 12 h of incubation, no presence of the metal was detected. In all cases, *P. aeruginosa* successfully degraded the metal salt, bioremediating the tested water. The presence of the PCL/Glu films, on the other hand, did not appear to enhance the removal of Cr (VI) by the bacteria. It is clear that the presence of the 3D structure of the fibers aided the bacteria to metabolize the metal faster. The degradation rate (mg day^−1^) was calculated using the degradation rate obtained at 8 h; the PCL/Glu fibers showed a degradation rate of 1.31 mg day^−1^, while PCL/Glu films have an efficiency of 1.14 mg day^−1^ and pristine *P. aeruginosa* has 1.07 mg day^−1^, respectively.

## 4. Discussion

### 4.1. Fourier Transform Infrared Spectroscopy

As observed in Figure 1A, none of the characteristic peaks of sugars can be detected; monosaccharides typically displayed two absorption peaks ν(O-H), in the range of 3520–3400 and 3192–3396 cm^−1^, while lactose, on the other hand, shows a broad ν(O-H) due to hydrogen bonding at 3522 cm^−1^, and additionally, saccharide sugar bonds can be seen at C-O stretch vibrations at 1077 and 1062 cm^−1^, C-C-H, O-C-H and C-O-H deformations at 1268, 1124 and 1062 cm^−1^, and another typical deformation can be seen at 624 cm^−1^ [34]. In all samples, only the peaks for the PCL control are seen, specially the stretching ν (C=O) [33]. 

For the scaffolds with nitrogen sources, we observed the latter behavior; no identifying peaks can be seen for glycine and urea. i.e., the two peaks corresponding to the symmetric and asymmetric vibration of ν(NH_2_) between 3426–3428 and 3327–3331 cm^−1^ are not observed [35]. Urea bonds and amide groups like the ones in peptone can be seen at absorption bands at 1630, 1550 and 1250 cm^−1^ [36,39], and lastly, the yeast extract has different regions in the spectra – because of its complexity, one region is characteristic of polysaccharides: “the sugar region” (950–1200 cm^−1^) and “the anomeric region” (750–950 cm^−1^), and protein residues can be seen at 1400–1700 cm^−1^ [40]. The low concentrations of Cs and Ns make it difficult to visualize spectra bands by FTIR, meaning further studies may be required. 

### 4.2. Thermogravimetric Assay and Differential Scanning Calorimetry

Thermal characterizations (TGA and DSC) were performed in this study, with the purpose of obtaining evidence of the presence of carbon and nitrogen sources on the PCL matrix by observing the mass degradation vs. temperature increment (TGA) and key temperature fluctuations (DSC) [19,41,42,43,44,45,46,47,48,49,50,51]. The calculated critical degradation temperature (TGA) is close to our reported decomposition temperature (DSC) of around ~380–390 °C. Degradation of PCL starts around ~330–340 °C, a behavior previously described by several authors [19,41,42].

With respect to the general appearance and classification of the TGA curve, all samples showed a desorption/drying curve where a large mass loss is followed by mass plateau [51]. In this assay, three key temperatures are usually important to measure: onset degradation temperature where the material starts to lose weight critically; midpoint or critical degradation temperature where the material loses 50% of its weight; and completion or decomposition temperature, where total mass loss is observed [51].

TGA applications range from obtaining the thermal and oxidative stability of the tested material, estimating life time or determining the effect of reactive or corrosive atmospheres on materials, although our purpose is to evaluate the incorporation of Cs and Ns on the PCL matrix, in order to assess the differences in composition in this multicomponent system, and thus the behavior of the materials needs to be sufficiently different on the temperature scale, to be able to identify it in the thermogram [51]. In this sense, all PCL/Cs and PCL/Ns fibers were compared with a pristine PCL fiber, since the purpose of this study was to recognize differences in thermal behavior of all the samples. A high temperature scan was employed (until 600 °C) to reach the complete degradation of the materials, and we observed any changes.

As observed in TGA thermograms of the fibers (Figure 2), small variations can be detected in the critical degradation temperature of the scaffolds. In the case of the Cs, a small increment was observed (~4 °C), while some of the Ns showed a slight temperature decrease (<~4 °C); this temperature increment in the Cs enriched fibers is probably due to the carbon bond rings, and the temperature decrement of Ns is possibly provoked by the presence of amino groups, since a previous report denoted that their presence caused a faster material decomposition [52]. Moreover, these small temperature fluctuations are expected to be due to the low proportion (0.16%) of the Cs and Ns added to the polymeric solution, but nonetheless some variations were still identified, giving evidence of the presence of Cs and Ns on the fibrous scaffolds. 

On the other hand, analyzing the DSC thermogram, similar thermal fluctuations can be seen [19,41,42], while previous studies shown a melting point of PCL around 60 °C [43], possibly indicating not only the melting of the material, but also the Cs present in the fibers, given the low temperatures at which these phenomena occur. The next relevant event in the thermogram arises at 360 °C, indicating the decomposition phase of the material, which has already been reported [44], reaching a peak in all samples at approximately 398 °C, with small variations between samples.

As reported earlier, lactose has a degradation peak at 220 °C and a melting point of 200 °C, surpassing dehydration at 145 °C [44]; glucose, on the other hand, has a degradation point of 339 °C and endothermic peaks at 227 °C and 305 °C [45], while galactose and fructose haven’t been reported in the literature. Nonetheless, it has been described that the thermal stability of the protein is enhanced when a sugar is added [46], which in turn could indicate the increase of the thermal properties of the fibers that contain Cs; additionally, disaccharides Cs like lactose increased the thermal properties of the fibers in a more superior way than monosaccharides Cs like fructose do. Likewise, Ns like peptone-enriched fibers have a degradation point of 213.31 °C and an endothermic peak at 197.14 °C [47], while glycine also has an endothermic peak at 195°C and experiences irreversible weight loss at 242 °C (same temperature as melting point) and a melting point of 242 °C [48], and urea has been reported in MgO nano powders to increase the endothermic peaks of the composites [49,50]. Yeast extracts have not been reported. Thus, the latter results suggest through its mild temperature fluctuations evidence of the presence of the Cs and Ns over the PCL matrix.

### 4.3. Bacterial Cell Growth Assay

From our obtained results, similar cell growth rates were observed in a previously published study [17], where poly (acrylic acid) (PAA), PAA/Chitosan and PAA/Alginate fibers reduced the cell growth of *E. coli*. Despite the fact that different culture media and different polymers were used in both studies, results are similar, showing a dramatic reduction in *E. coli* cell growth compared to control samples.

*Moffa* et al. [18] showed that it is possible to optimize the yield of cultures in the production of secondary metabolites using electrospun scaffolds from *S. lividans*. The report indicated that the strain used in their experiments seemed to prefer a fibrous support, rather than the glass support offered by the flask, possibly using the former scaffolding as an anchor site, where it can generate mycelia. The mobility of *S. aureus* is known to be reduced compared to *E. coli* and *P. aeruginosa*, though mobility amongst the strains may contribute in some way to the performance of the cultures in the fibers, although it should also be taken into account the effect of porosity, as in the growth of *P. aeruginosa* [53] and how the possible adjustment of this parameter could lead to crops that may have the same potential as *S. aureus*. 

In this work, PCL fibers were chosen to be enriched with carbon and nitrogen sources, as PCL fibers have been reported to have a minimal effect on bacterial cell growth [17,53,54,55,56,57]. From the tested bacteria in this study, PCL fibers did not elicit any decrease in cell growth for *P. aeruginosa* (Figure 5), as observed in a previous report, where the cell growth of *P. aeruginosa* and *S. epidermidis* was not affected with PCL fibers after 6 h of incubation [17]. Moreover, this result is similar to the obtained by Ramírez-Cedillo et al., which reported that PCL fibrous scaffolds did not alter the cell growth of *S. aureus* [53]. In contrast, our results (Figure 6) showed a 14% decrease in cell growth in *S. aureus* at 48 h, with this behavior continuing after 72 h, while *P. aeruginosa* exposed to PCL fibers proliferated quickly while producing extracellular polymeric substances (EPSs), where it has been demonstrated that the first hours of incubation are important for avoiding biofilm formation [55].

In general, for the Gram-negative tested bacteria (*E. coli* and *P. aeruginosa*), the PCL samples independent of their structure (films or fibers) did not show a significant decrease (ANOVA *p* < 0.05) or enhancement of the bacterial cellular growth. Some reports explained that this type of bacteria possesses better protection against antimicrobial agents, due to their cell wall-specific molecules; in this sense, lipopolysaccharides, which are molecules present in the bacterial cell wall, provide permeability to the cell against several antimicrobial agents, and slow the influx of lipophilic compounds. On the other hand, the porin proteins embedded in the cell membrane give the principal channels for the entry of solutes into the cells [58].

In our previous work [57], we compared the capacity of biofilm formation using poly (caprolactone)/curcumin (PCL/CUR) fibers and films. In the study, it was demonstrated that PCL/CUR fibers significantly improved the biofilm formation of *S. aureus* and *E. coli* at all times tested (24, 48 and 72 h). In contrast, our current work showed that *P. aeruginosa* did not present an interesting biofilm formation, except at 24 h, where the cell growth of the bacteria was improved. PCL/CUR films were not as effective, and hindered the cell growth rate of all the tested bacteria. In the aforementioned study, the enhancing properties for biofilm formation of the PCL/CUR fibers was attributed to the tridimensional architecture that the fibers created, increasing the high surface area contact [8]. 

### 4.4. Biofilm Formation Observation by Scanning Electron Microscopy (SEM)

Despite the fact that many of the PCL/Cs and PCL/Ns fibers samples did not show significant bacterial growth (ANOVA *p* < 0.05) in the “bacterial cell growth assay”, this is not a determinant factor in the biofilm formation capacity of the scaffolds; since the former method just quantifies the bacterial cells suspended in media when the reading is taken by the spectrophotometer, it is important to consider the proportion of bacteria that adheres to the fibrous scaffolds. As reported by Himmler et al. [58], a cell-scaffold interaction assay is still needed, where a known dimension of the fibrous scaffold is seeded with a known number of cells. After a certain time of incubation, non-adherent cells are removed by rinsing the fibrous scaffold, and the retained cells are stained with a specific dye; after the micrographies are obtained, cell density is quantified by counting the cell numbers in a particular area using Image J software.

From the tested fibers samples, we chose the PCL/Glu fibers to better visualize biofilm formation over and between the fibers. As observed in Figure 7, bacterial cell replication took over on the surface and within the fibrous scaffolds, mainly due to the porosity (44.31%) of the scaffolds, which permits bacterial cell penetration into the frames. The latter is supported by comparing the cell size of each tested bacteria; *E. coli* possesses a cylinder dimension of 1–2 µm in length, with a radius of 0.5 µm [59,60]. For *P. aeruginosa*, the reported cell size is around ~10 μm [38,57,61] and *S. aureus* is between 1–1.5 μm in length, respectively [57,58,62]. Taking into account our obtained porosity and that which was reported in the literature, PCL scaffolds usually presented a pore size of around 10–45 nm [57,58,63]. Moreover, when the bacterial cells proliferate inside the mat, their limited movement causes cell stress, which in turn results in the synthesis of extracellular molecules that fill all the present pores in the scaffolds, creating a well-defined biofilm which can be transported and further processed. Another possible phenomenon can be discussed due to *P. aeruginosa* cells’ size; when cells grow and replicate only on the surface of the fibrous scaffolds, the synthesized biofilm products were found between the fibers, creating the thick mass shown in Figure 7G,I. This latter behavior was observed by Himmler et al. [58] when they grew corneal endothelial cells on fibrous polycaprolactone (PCL), PCL/collagen, PCL/gelatin and PCL/chitosan scaffolds, and observed a superficial cell attachment over the fibers and replication. Despite all reported evidence, further analysis in the bacterial biofilm formation in the fibrous scaffolds is warranted still.

From our results, the obtained fiber diameter ranged from 313 to 766 nm, where most of the sample’s range between 400 and 500 nm. Our obtained fibers were similar to those reported by Ruckh et al. [54], where the PCL fiber diameter was between 557 ± 399 nm. Many PCL fiber diameters have been reported for biomedical applications, which is the most intended approach of PCL fibers, such as Ruckh et al. [54], which obtained PCL fibers of 557 ± 399 nm, proposed for bone tissue engineering and for drug release of rifampicin. In addition, Yang et al. [63] reported 120 nm’s PCL fibers and Ramírez-Cedillo et al. [53] PCL fibers with a higher diameter such as ~1000 nm, approximately, which were also proposed for bone tissue regeneration. As noted, a great variability of fiber diameter can be encountered for the PCL fibers.

As mentioned before, the aim of incorporating a tridimensional structure by using inert polymeric nanofibers such as PCL is to stimulate bacterial biofilm formation by provoking cellular stress in the exposed bacteria [64]. In that sense, Chu et al. [65] discussed the mechanical stress on bacteria which stimulate biofilm formation; these mechanisms arise first with physical stress associated with colony confinement in the pore area of the scaffolds [66]. Subsequently, the biofilm creation can be supported by a modulated nutrient access, where the biofilm matrix deposition is higher and mechanical and biological stresses are present, and the accumulated matrix further increases the stress levels. Moreover, a feedback regulation can lead to cellular adaptative responses from the bacteria creating the EPSs [57]. Thus, our PCL/Cs and PCL/Ns tridimensional scaffolds mean to generate the above mechanical stress to bacterial cells. 

Hence, since *E. coli* and *P. aeruginosa* need require cellular motility for their replication process [67], the use of electrospun fibers to block the movement of the bacterial cells is highly coherent, and further studied. In addition, under stressful conditions, bacterial cells can actively pursue a protective, spatially isolated niche, such as microcavities in the complex mechanical microenvironments or the cytosolic compartments of the bacterial cells. By creating the biofilm, colonies can be shielded from adverse stimuli coming from the environment and proliferate in high degrees. The resultant aggregated colonies lead to various forms of collective behavior, shaped by mechanical and biological properties [57,66].

For our proposed application, it is desirable that fibers work as a transporter for biofilms which are projected to be used in bioremediation applications such as heavy metal removal in water. Primarily, this study intends to evaluated if any of the PCL/Cs or PCL/Ns scaffolds are suitable to produce controlled bacterial biofilm. Still, further studies are necessary to optimize the fibrous systems in order to find the adequate fiber diameter and morphology (Figure S1), as well as the desired mechanical properties of the systems.

In the case of an optimized morphology and fiber diameter, an adequate viscosity property needs to be studied in samples where a suitable bacterial growth was observed, such as PCL/Gal fibers for *E. coli*, PCL/Glu fibers for *P. aeruginosa* and PCL/Lac and PCL/Gly fibers for *S. aureus.* These latter formulations will be studied, and an optimization of the electrospinning parameters will be improved. In that sense, the morphology and fiber diameter are highly dependent on the viscosity of the solution and conductivity of the chosen solvent, as well as determining an adjusting polymer concentration in order to obtain the best quality of the fibers [8,10]. The fiber diameter needs to be balanced with the desired mechanical property for the above-discussed application, in order to decide which fiber diameter fits with the intended property of the scaffolds. As seen in Figure 7, optimized PCL/Glu fibers were prepared and no bulbs were observed. This was achieved by adjusting parameters on the electrospinning process where a variation of the voltage and adjusting the environmental parameters, such as the % humidity, improved the fibers’ formation without changing the polymer concentration or the solvent [8,57]. In this work, a 13% solution of PCL in THF was prepared. Similar concentrations have been previously reported for the successful preparation of fibers [56,57,68]. Moreover, studies have reported that chloroform, as well as THF, have been used to successfully electrospun PCL fibers. We chose THF due to its miscibility in water [68], as well as better compatibility with the carbon and nitrogen source solution.

### 4.5. Chromium Removal Assay

The contamination and accumulation of heavy metals is a worrying phenomenon, and furthermore, the chemical processes used for their treatment are expensive and often harmful to the environment; therefore, an interest in developing methods that facilitate their elimination from the environment without damaging it or without an adverse effect are being researched. The ability of some microorganisms to use, degrade and remove heavy metals from the environment is being used, together with methods of immobilization of the microorganisms on biofilms on a suitable support in bioreactors. For example, biofilms of bacteria such as *Pseudomonas putida* or *Chromobacterium violaceum* have been used for the biodegradation of pesticides such as cypermethrin, with a biodegradation of up to 90% [69,70].

The applicability and benefits that the use of biofilms can have on different fields of bioremediation, due to their resistance and versatility, increases the need to research them, as well as to know them in their fields of action in order to provide favorable solutions to current environmental problems [69,70].

Quantifying the amount of biofilm production in further studies owes its interest to its potential use in a safe bioremediation approach. Biofilms permit the sessile cells to live in an organized, more permanent manner, which supports their propagation. The biofilm is a surrounding matrix that mostly includes exopolysaccharides, which allows their adherence to inert or organic surfaces [71].

Chromium removal in water by *P. aeruginosa* has been reported extensively [70,71,72,73,74,75,76,77,78,79], but to the best of our knowledge, no reports are found in the literature that use electrospun fibers as promoters of intended *P. aeruginosa* biofilm formation for the removal of chromium from water, and thus a comparison between the reported bioremediation potential of *P. aeruginosa* biofilms created with non-nanostructured substrates and our results is listed in Table 5, in order to see the difference in the removal capacity with and without the use of nanostructured substrates.

As observed, our results are similar to Mat Arisah, F. et al. 2021 [72], which achieved an 82% removal efficiency at 8 h, just as our enhanced biofilm of *P. aeruginosa* incubated in PCL/Glu scaffolds, which increases this reported efficiency up to 87%. Our pristine *P. aeruginosa* and PCL/Glu films/*P. aeruginosa* were not as efficient, but still higher than other studies [72,74]; this can be due to the use of extra nutrients such as the PCL and Cs and Ns sources. 

Ozturk et al. [71] explained that Cr (VI) can be reduced to Cr (III) in aqueous solution by direct contact with the electron-donor groups of the biomass. On the other hand, this reduction can be due to the union of the anionic Cr (VI) ion to the positively charged groups existent on the biomass, or the reduction can be realized by Cr (III) ion release in to the aqueous phase, due to the electronic repulsion between the positively charged groups on the cells surface and the Cr (III) ions. Moreover, the capacity to reduce Cr (VI) and to resist high Cr (VI) concentrations were found to be independent properties of bacteria, especially *Pseudomonas* sp. [72]. 

Another study discussed that Cr (VI) ion can be reduced by an extracellular sequestration mechanism. This sequestration is considered to be enabled by the production of membrane-associated bioproducts, such as extracellular enzymes and biosurfactant; these metabolites are prevalent in the *Pseudomonas* family. Moreover, as more metabolites are produced, a lesser amount of Cr (VI) is absorbed by the bacteria. The secreted metabolites readily reduced the sequestered Cr (VI) to Cr (III) ions in the extracellular space. This avoids the penetration of the ions into the cell membrane, since Cr (III) cannot readily diffuse into the cell membrane as Cr (VI) ions do [73,74]. 

Further work is necessary to assess the amount of biofilm formed into the fibers and films; i.e., following and adapting a study by Himmler et al. [58], where a cell-scaffold interaction assay was reported; this test can be modified for bacterial biofilms with the purpose of quantifying the proportion of bacterial cell attachment to the fibers, thus comparing the efficiency of both types of scaffolds (anchor sites); moreover, it is important to determine the mechanism of action of the chromium uptake in aqueous solution. Furthermore, it is necessary to repeat the chromium removal experiment, with the purpose of adding more intermediate readings points, to determine the exact time for total chromium removal. Lastly, additional research into the development of methods for drying the formed biofilm on the fibrous scaffolds is warranted, in order to create a biofilm transport system to be performed in situ bioremediation of contaminated water.

## 5. Conclusions

The effect of the presence of PCL fibers on microbial cultures was evaluated; *E. coli* does not show a favorable response when fibers were added to the medium, since its growth diminishes with time. Both *P. aeruginosa* and *S. aureus* show an increase in bacterial concentration of 95% and 53% at 48 h, compared to the control culture. In fibers, bacteria seem to have a normal development; when they grow on a surface they begin to adhere to the fibers, or are fixed to the pores of the material and establish small colonies that, according to the possibilities of movement of the bacteria, disperse throughout the material and begin to generate a biofilm on the fibers. The relationship that exists between the fibers and the generated biofilm has yet to be established, which seems to be more sensitive to the chemical composition of the polymer. Despite all the clear advantages of the use of fibrous scaffolds for biofilm formation, no clear difference for enhancement can be observed between both structures (films and fibers); however, PCL/Glu fibers were successful in increasing the bacterial growth of *P. aeruginosa* to ~36%. This system was used to evaluate Cr (VI) removal in aqueous solutions, which showed enhancement with an 16% efficiency higher than the pristine bacteria.

## Figures and Tables

**Table 1:** PCL/Cs and PCL/Ns fiber and film composition.

Sample Label	Cs ^a^ Solutions	Sample Label	Ns ^b^ Solutions
PCL	PCL (Control)	PCL	PCL (Control)
PCL/Glu	PCL and Glucose	PCL/Pep	PCL and Peptone
PCL/Lac	PCL and Lactose	PCL/Gly	PCL and Glycine
PCL/Fru	PCL and Fructose	PCL/Yea	PCL and Yeast Extract
PCL/Gal	PCL and Galactose	PCL/Ure	PCL and Urea

[^a^] Cs: Carbon sources; [^b^] Ns: Nitrogen sources.

**Table 2:** % Weight loss of PCL/Cs and PCL/Ns fibers vs. temperature increment.

Sample	Onset Degradation Temperature (Ti) 5%	Critical Degradation Temperature 50%	Final Degradation Temperature 100%	Sample	Onset Degradation Temperature (Ti) 5%	Critical Degradation Temperature 50%	Final Degradation Temperature 100%
PCL	336.23 °C	388.39 °C	412.96 °C	PCL/Pep	342.86 °C	386.89 °C	570.56 °C
PCL/Glu	347.51 °C	393.64 °C	418.74 °C	PCL/Gly	333.24 °C	388.38 °C	417.52 °C
PCL/Fru	341.56 °C	392.50 °C	416.46 °C	PCL/Yea	305.02 °C	384.62 °C	427.38 °C
PCL/Lac	349.87 °C	392.48 °C	417.08 °C	PCL/Ure	273.16 °C	389.51 °C	548.68 °C
PCL/Gal	346.33 °C	392.35 °C	416.20 °C				

**Table 3:** Characteristic temperatures of differential scanning calorimetry.

Sample	Melting Temperature	Decomposition Temperature	Sample	Melting Temperature	Decomposition Temperature
PCL	63.55 °C	397.88 °C	PCL/Pep	62.92 °C	395.64 °C
PCL/Glu	62.44 °C	398.79 °C	PCL/Gly	61.02 °C	396.62 °C
PCL/Fru	61.99 °C	398.28 °C	PCL/Yea	61.54 °C	393.37 °C
PCL/Lac	61.61 °C	397.28 °C	PCL/Ure	60.73 °C	395.54 °C
PCL/Gal	61.97 °C	398.32 °C			

**Table 4:** Average fiber dimeter with standard deviation (SD) for PCL, PCL/Cs and PCL/Ns fibers.

Sample	Average ± SD (nm)	Sample	Average ± SD (nm)
PCL	581 ± 129	PCL/Pep	480 ± 146
PCL/Glu	313 ± 089	PCL/Gly	473 ± 124
PCL/Fru	558 ± 162	PCL/Yea	766 ± 290
PCL/Lac	301 ± 072	PCL/Ure	332 ± 083
PCL/Gal	408 ± 143		

**Table 5:** Chromium (VI) removal from aqueous solution by *P. aeruginosa.*

Organism	Time of Incubation (h)	Removed Chromium (%)	Reference
*P. aeruginosa*	48	24	[72]
*P. aeruginosa 99*	48	45	[72]
*P. stutzeri T3*	48	33	[72]
*P. aeruginosa RW 9*	8	82	[73]
*P. aeruginosa P16*	45	37	[74]
*P. aeruginosa 4442*	8	22	[75]
*P. aeruginosa*	8	71	*Our study*
PCL/Glu fibers/*P. aeruginosa*	8	87	*Our study*
PCL/Glu films/*P. aeruginosa*	8	76	*Our study*

## Supplementary Materials

The following supporting information can be downloaded at: https://www.mdpi.com/article/10.3390/membranes12030327/s1, Figure S1: PCL, PCL/Cs and PCL/Ns samples fibers. (A) PCL, (B) PCL/Glu, (C) PCL/Fru, (D) PCL/Lac, (E) PCL/Gal, (F) PCL/Pep, (G) PCL/Gly, (H) PCL/Yea, (I) PCL/Ure. All micrographs were taken at 1000× of amplification and 20 µm of reference scale for measuring.
