# Supplementary material for: Comparative Study of Polycaprolactone Electrospun Fibers and Casting Films Enriched with Carbon and Nitrogen Sources and Their Potential Use in Water Bioremediation
Source: Membranes (Basel). 2022 Mar 15;12(3):327. doi: 10.3390/membranes12030327 (PMC8951516; doi:10.3390/membranes12030327)
Supplement: Supplementary file 1 [file membranes-12-00327-s001.zip › membranes-1561122-supplementary.pdf]

membranes

Supplemental materials

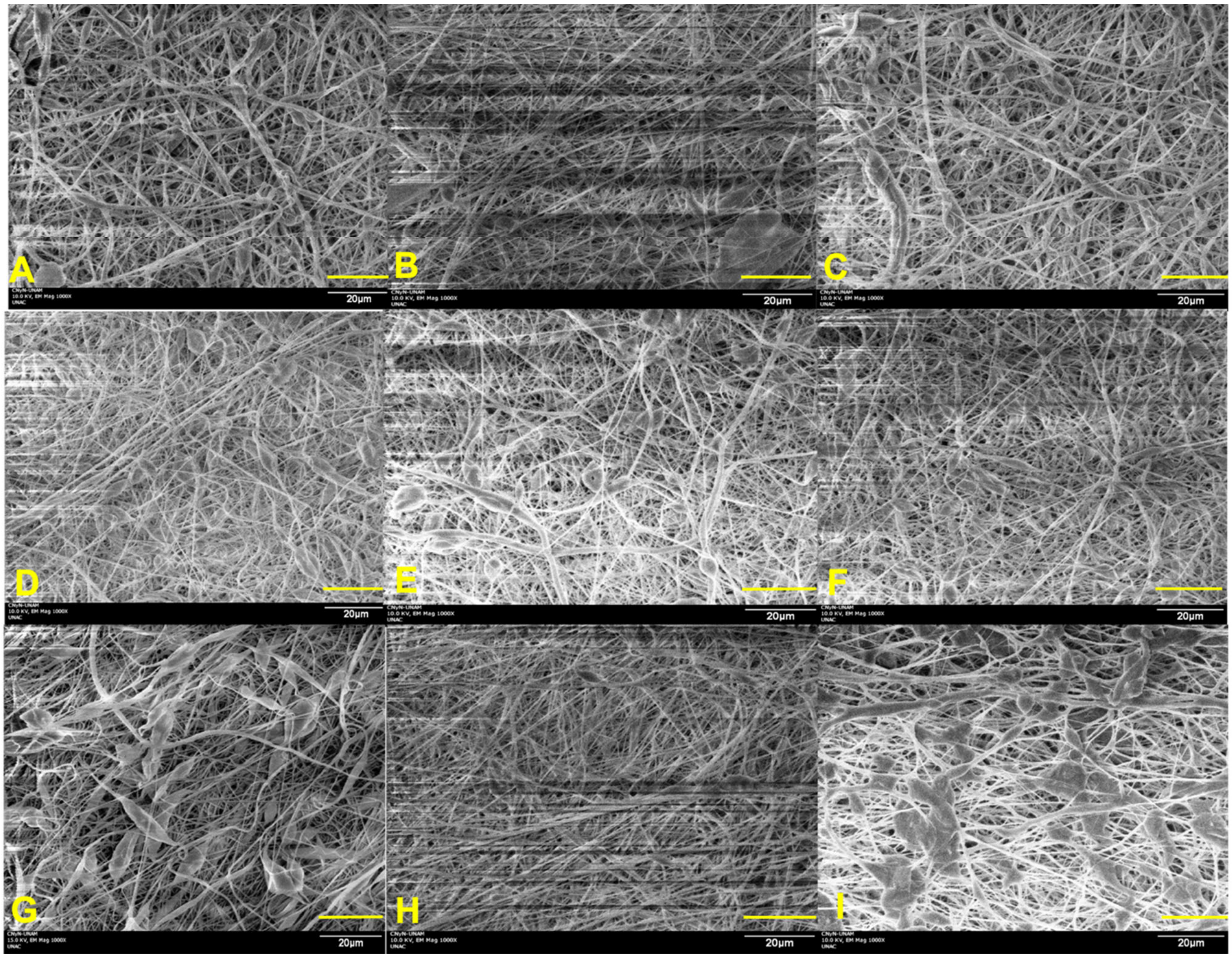

Figure S1. PCL, PCL/Cs and PCL/Ns samples fibers. A) PCL, B) PCL/Glu, C) PCL/Fru, D) PCL/Lac, E) PCL/Gal, F) PCL/Pep, G) PCL/Gly, H) PCL/Yea, I) PCL/Ure. All micrographs were taken at 1000 x of amplification and 20 μm of reference scale for measuring.
